# Analysis of Trunk Rolling Performances by Mattress Mobility Detection System in Poststroke Patients: A Pilot Study

**DOI:** 10.1155/2016/8743051

**Published:** 2016-03-03

**Authors:** Shang-Lin Chiang, Chia-Huei Lin, Chueh-Ho Lin, Liang-Hsuan Lu, Shin-Tsu Chang, Wen-Hsu Sung, Shun-Hwa Wei

**Affiliations:** ^1^Department of Physical Medicine and Rehabilitation, Tri-Service General Hospital, National Defense Medical Center, Taipei, Taiwan; ^2^Department of Physical Therapy and Assistive Technology, National Yang-Ming University, Taipei 114, Taiwan; ^3^Department of Nursing, Tri-Service General Hospital, National Defense Medical Center, Taipei, Taiwan; ^4^School of Gerontology Health Management and Master Program in Long-Term Care, College of Nursing, Taipei Medical University, Taipei, Taiwan; ^5^School of Medicine, National Defense Medical Center, Taipei, Taiwan; ^6^Department of Physical Medicine and Rehabilitation, Taichung Veterans General Hospital, Taichung, Taiwan

## Abstract

*Purpose. *The purpose of this study was to investigate the correlation of kinematic variables with quality of trunk control in poststroke patients.* Methods. *This cross-sectional study included stroke subjects with mild to moderate motor deficit corresponding to Brunnstrom stages 3-4. Trunk functional performance was measured using bed mobility monitor system. All tasks were repeated ten times for both directions in each subject. Outcome measurements included the movement time and displacement of center of pressure (CoP) from supine to side lying and returning.* Results. *The results revealed that a significant longer turning time was observed when turning from the paretic side toward the nonparetic side compared to the other direction, with an estimated mean difference of 0.427 sec (*P* = 0.005). We found a significant difference in the time of rolling back to supine position between two directions. The displacement of CoP in rolling back from side lying on the nonparetic side was smaller than that from the paretic side with an estimated mean difference of −0.797 cm (*P* = 0.023).* Conclusions. *The impaired trunk mobility was associated with increased movement time and decreased displacement of CoP in poststroke patients. Trunk rolling performance has potential in assessment of stroke patients.

## 1. Introduction

Stroke is one of the predominant causes of long-term disability worldwide. Stroke-related impairments contain cognitive, language systems, visual, sensory, and motor. Among fifty percent of stroke survivors, chiefly aged and middle-aged people undergo hemiparesis, which is a muscle strength insufficiency caused by nervous system injury of brain. Patients with hemiparetic stroke display muscle weakness, abnormal posture and muscle tone, loss of coordination of trunk and limb movements, and impaired trunk control [1, 2]. In poststroke patients, impairment of upper limb function has been exposed to be a forecaster in quality of life and activities of daily life [3]. In addition to extremity function, hemiplegia has effects on the function of trunk muscles on both sides of the body affecting the proximal control [4]. Several instruments have been developed for the assessment of stroke patient in rehabilitation view; however, observation and subjective measurements are employed.

Trunk muscles play a major role in maintaining antigravity postures and in stabilizing body during voluntary limb movements. Impairment of trunk muscles strength in stroke is associated with balance difficulty in poststroke patients [2, 5, 6]. Trunk performance has been demonstrated to be a predictor for the functional outcomes after stroke. In patients with hemiparetic stroke, the length of hospital stay is associated with trunk function [7, 8]. Trunk performance in sitting position after stroke predicted functional ability and destination at discharge from rehabilitation [9]. Instruments designed to assess trunk functions in stroke patients include Functional Independence Measure (FIM), Fugl-Meyer balance test, Postural Assessment Scale for Stroke Patients (PASS), and Trunk Control Test (TCT). Among these, TCT has been the most commonly used instrument, which examines the maintenance of the sitting position, the ability to roll from a supine position towards the paretic and nonparetic sides, and the transfer from supine to sitting position. Although these scales are useful for clinical assessment, the changes in scores are difficult to interpret.

Recent studies have reported several approaches to assess trunk impairment in stroke patients, measuring muscle strength, electromyographic activities or motor-evoked responses elicited with transcranial magnetic stimulation of trunk muscles. However, correlation of kinematic variables with quality of trunk control in poststroke patients remains sketchy. We hypothesized that stroke patients with impaired trunk function exhibit different rolling patterns on bed mobility monitor system. Thus, the effects of trunk rolling performances on trunk control in poststroke patients were investigated by the bed mobility monitor system developed for quantitative measurements.

## 2. Methods

### 2.1. Subjects

In this cross-sectional study, seventeen patients were recruited from the Department of Rehabilitation at Taipei Tri-Service General Hospital. Study protocol was reviewed and approved by the Institutional Review Board, Taipei Tri-Service General Hospital. All subjects signed informed consent before enrollment in this study. Eligible poststroke patients met the following inclusion criteria: (1) infarction or hemorrhage type cerebral vascular accident (CVA), (2) first time stroke without any severe complicated disease, impacting the trunk movement, (3) at least 6 months since stroke and stability, (4) the Brunnstrom Stage should be three or higher, and (5) Mini-Mental State Examination (MMSE) score should be 24 or higher. Exclusion criteria included (1) unstable cardiovascular condition, (2) uncontrolled hypertension (190/110 mm Hg), (3) severe orthopedic or pain conditions, (4) aphasia with inability to follow researcher's commands, (5) severe joint contracture of upper or lower extremities that would impact the trunk movement performances, (6) severe obesity, or (7) Ashworth scale ≥3.

### 2.2. Equipment

**Figure 1:**
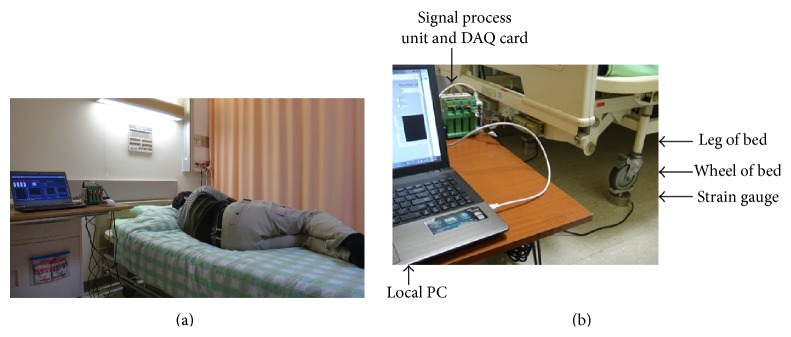
(a) Trunk rolling performance was assessed using bed mobility monitor system. (b) Bed mobility monitor system. There were sensors attached to the bed leg.

The bed mobility monitor system was employed, which was designed to allow the subject to perform the trunk functional performance on bed [10] (Figure 1). In brief, four strain gauges (LFS1CC 150 kg, Delta Transducers Co.) were mounted under the feet of bed and were used to evaluate the center of mass and center of pressure during task executing. All sensors were connected to a laptop computer via a 12-bit analog-to-digital converter to the LabVIEW 2010 (National Instruments Corporation, Austin, TX, USA) data acquisition system. Data was managed and analyzed through data interception using a software program written using LabVIEW, 2010 (National Instruments Corporation, Austin, TX, USA) to identify or distinguish the trunk performances, such as rolling and sitting up or off the bed. The sampling frequency was set at 10 Hz.

### 2.3. Testing Protocols and Study Flow

Each subject was asked to roll in the side toward paretic or nonparetic side as fast as possible after hearing the researcher's command and then remained on side lying position for 10 seconds and rolled back to supine position as fast as possible after hearing the researcher's command. Ten trials were executed with 1 minute resting interval (to avoid muscle fatigue) for each paretic or nonparetic side (randomized assign).

### 2.4. Outcome Measurements

The changes in CoP were measured and analyzed as movement time, distance, and peak pressure of counteraction of trunk rolling performance. The movement time of rolling performance was defined as the operation time starting from the movement of trunk or limbs toward the paretic or nonparetic sideway. Main outcomes of the study included side turn time, movement distance of CoP, velocity of CoP, peak pressure of counteraction, time to reach peak pressure, and the ratio of turn peak to turn time (swiftness) (turn time/Back time, TL/BL, turn/back speed, turn/back force, turn/back peak time, and turn peak/back peak/time).

### 2.5. Statistical Analysis

Continuous variables were summarized by means and standard deviations, ordinal variables (MMSE, Brunnstrom stage (BS), PASS, TCT) were summarized by medians and full ranges, and categorical variables were presented by counts and percentages. The paired *t*-tests were performed to compare the turning ability between two turning directions (toward the nonparetic side versus toward the paretic side), based on the averaged turning ability parameters of the 10 repeated measurements of each subject. The factors associated with turning ability parameters were performed by using the generalized linear model (GZLM) with identity link function, where the generalized estimating equation (GEE) with exchangeable working correlation matrix was applied for the repeated measurements. GZLM models were performed, respectively, for two rolling directions: rolling from supine to side lying position and rolling back from side lying position to supine. When there are two or more factors with *P* value less than 0.1 in the univariable models, the factors would be entered into the multivariable model for adjustments. Two-sided *P* value less than 0.05 indicated statistical significance. The statistical analyses were assessed by the software IBM SPSS Statistics 19.0 (IBM Corporation, Armonk, New York).

## 3. Results

### 3.1. Demographic Data

A total of 17 poststroke patients were recruited, including 11 males and 6 females, with a mean age of 63.9 years. Of the subjects, 14 and 3 were diagnosed with stroke within 1 year and 1-2 years, respectively. The other demographic details were summarized in Table 1.

Turning ability between the two directions towards the paretic and nonparetic side was determined.

According to the results of the paired *t*-tests (based on the averaged turning ability parameters of the 10 repeated measurements of each subject), no significant difference were observed in other parameters (Table 2).

Use of GZLM models showed that, with or without adjustments for other covariates, factors associated with turning ability parameters were as follows: (i) turning toward the nonparetic side took significantly longer turn time (mean difference of 0.427 sec, *P* = 0.005) and longer time to turn peak (mean difference of 0.48 sec, *P* < 0.001); turning toward the nonparetic side had longer turn peak/time (turning swiftness) (mean difference of 0.069, *P* = 0.001), respectively; (ii) turning back from side lying on nonparetic side took less time compared to the other direction from side lying on paretic side (mean difference of −0.252 sec, *P* = 0.044) and had less BL (mean difference of −0.797 cm, *P* = 0.023); compared to the other direction (from side lying on paretic side), turning back from side lying on nonparetic side had higher back force (mean difference of 1.234 kg·m/s^2^, *P* = 0.002) and higher back force/weight (mean difference of 0.022, *P* = 0.001); (iii) patients with Brunnstrom stage of upper extremities (BSUE) ≤3 had higher turn peak/time ratio (mean difference of 0.085, *P* = 0.021); (iv) patients with Brunnstrom stage of lower extremities (BSLE) ≤3 took longer back time (mean difference of 0.536 sec, *P* = 0.003) and longer time to peak counteraction when rolling back (mean difference of 0.492 sec, *P* = 0.004) and had longer back peak/time ratio (mean difference of 0.058, *P* = 0.032), respectively; (v) patients with PASS ≤18 had lower turn peak/time ratio (mean difference of −0.089, *P* = 0.009); (vi) patients with TCT ≤50 took less time in back peak (mean difference of −0.569 sec, *P* = 0.004) and back peak/time (mean difference of −0.076, *P* = 0.017), respectively (Tables 3 and 4).

## 4. Discussion

In the present study, we demonstrated that poststroke patients exhibited significant difference in rolling performance from supine position toward paretic and nonparetic sides. CoP displacement during trunk back from the paretic side toward the nonparetic side was significant smaller than turning back from the other direction. We found that the movement time for rolling back from side lying on the nonparetic side was significant less than rolling back from side lying on the paretic side. It is indicated that stroke patients exhibited perturbed rolling pattern in comparison with healthy subjects [10].

Trunk rolling is considered to play a fundamental function in most daily activities [11]. Rolling from supine to side lying represents a key step in movement development [12]. Recent studies have shown that the trunk rolling performance on bed can be used as an important predictor to predict the walking ability for 1.5-year children with cerebral palsy [13, 14]. Trunk rolling movement is important in intersegment coordination control between limbs, and head and trunk are controlled by brain [15–17]. People with neurologic deficits such as stroke exhibit reduced muscle strength, paresis, spasticity, and discoordination of hemiparetic side as well as decreased sensorimotor integration of central nerve system [18, 19]. Additionally, hemiparetic stroke significantly impacts the walking ability, posture control, balance, and functional performances due to abnormal coordination control between limbs or compensatory trunk or limbs movements for subacute or chronic stroke patients [19–21]. In this study, we found sequence movement of body segments included head, upper extremity movements, and trunk rotation, which was consistent with previous study [22]. However, this sequence movement of body segments was not very clear and smooth. It is postulated with abnormal muscle spasticity or discoordination of hemiparetic limbs and trunk, resulting in longer movement time and smaller CoP displacement. Those were found during rolling toward the nonparetic side or shorter movement time and smaller CoP displacement during rolling back from side lying on the nonparetic side. It is suggested that the motor impairment of limbs or trunk would significantly impact the trunk rolling performances [10]. A recent study has shown the delay of movement time of upper extremity, head, and trunk among stroke patients [17]. Our data support this movement time delay as increasing movement time for rolling toward the paretic side and back from side lying on the paretic side. Consistent with previous study using videotape recording to analyze the rolling performances on bed, it is indicated that the movement of upper limb is the first movement during the trunk rolling performances from supine to side lying [12]. In an agreement with previous study, our result revealed that poor upper extremity function (BSUE ≤ 3) was associated with poor swiftness (turn peak/time). In addition, we found that the movement of head flexion was the first movement during the trunk rolling toward nonparetic side. Previous studies have shown that the head movement or upper extremity performance has no influence on the posture in sitting or standing and significantly impact the spatial and temporal characteristics during trunk rolling performance [17, 19, 21]. In this study, we found that hemiparetic stroke patients exhibited less movement time while rolling back from side lying on nonparetic side to supine position. This is supported by observation in clinical practice that poststoke patients failed to perform trunk rolling with impaired limbs with a result of dropping trunk after passing the center of mass. Additionally, we found that stroke patients exhibited less movement CoP while rolling back from side lying on nonparetic side. It is postulated that the failure of trunk control is attributed to muscle weakness and discoordination incorporating with psychological factor as returning to safe positon. We used the trunk rolling task as the 3D motor evaluation (rolling sideway from supine position) for trunk functional measurements for stroke patients, causing the spatial and temporal characteristics changes in CoP displacement and movement time that is effective to evaluate the functional trunk control.

Several measurement modalities have been developed to evaluate the trunk control performance, such as TCT, PASS, and TIS. Clinicians are dependent on such tools for making judgement and decision. TCT with 3-point scale is commonly used to measure the trunk control and predict the comprehensive activities of daily living [23]. TCT measure is implicated to predict the trunk functional performances in stroke with good correlation with the Functional Independence Measure [7, 24]. PASS, a scale-based measurement, is one of the high validity and reliability clinical measurement for posture control for people with stroke [25]. It has been reported that TCT and PASS were similar, but PASS used 4-point scores and contained 5 specific trunk control test, which could be more suitable for measuring the trunk functional performance than the TCT [26]. Recent study has shown the significant ceiling effect of PASS and TCT measurements for assessing the trunk control performances, impairments, or functional recovery for people with stroke [27, 28]. TIS is developed to evaluate the dynamic or static sitting balance and trunk coordination control [29]. In the present study, we demonstrated that trunk rolling performance was correlated with the results of two evaluation tools currently used in practice, TCT and PASS. It is suggested that trunk rolling performance assessed using the monitoring system has the potential to predict motor recovery and comprehensive activities of daily living. In addition, the monitoring system is expected to offer physicians an assessment tool that scientifically quantifies data over evaluation process rehabilitation course.

There are several limitations in this study. This study had a small population size. The bed mobility monitor system was unable to analyze each body segment movements severely (arms, legs, and head) and in relation to trunk, which could provide several information and suggestions for the rehabilitation. Further studies are required to examine the impact and relation between different motor recovery, clinical measurements, and functional trunk control mobility, as the predictor for trunk functional recovery, and developing appropriate rehabilitation programs, treatment goals, or designing home exercise to improve the trunk functional performance for people with stroke.

## 5. Conclusion

We concluded that the trunk mobility was declined as increased movement time and decreased displacement of center of gravity in stroke patients. These findings highlight trunk control deficit changes in poststroke patients. Trunk rolling performance has potential in assessment of stroke patients, providing information for design of an appropriate intervention and rehabilitation program for stroke patients.

## Figures and Tables

**Table 1:** Demographic data.

	*N* = 17
	
Age^1^ (year)	63.9 (13.2)
Gender^2^	
Female	6 (35.3%)
Male	11 (64.7%)
Paretic side^2^	
Left	9 (52.9%)
Right	8 (47.1%)
Height^1^ (cm)	164.3 (8.1)
Weight^1^ (kg)	67.4 (12.7)
BMI^1^ (kg/m^2^)	24.8 (3.4)
Duration of diagnosed stroke^2^	
<1 year	14 (82.4%)
1-2 year	3 (17.6%)
Diabetes^2^	
Yes	7 (41.2%)
No	10 (58.8%)
Hypertension^2^	
Yes	12 (70.6%)
No	5 (29.4%)
Heart disease^2^	
Yes	6 (35.3%)
No	11 (64.7%)
MMSE^3^	26 (22, 29)
BSLE^3^	4 (3, 6)
BSUE^3^	3 (2, 5)
PASS^3^	16 (10, 24)
TCT^3^	48 (38, 87)

^1^Data are presented by mean and standard deviation. ^2^Data are presented by count and percentage. ^3^Data are presented by median and full range. MMSE, Mini-Mental Status Examination; PASS, postural assessment scale for stroke patients; TCT, trunk control test; BSLE, Brunnstrom stage of lower extremities, BSUE, Brunnstrom stage of upper extremities.

**Table 2:** Comparison of turning ability parameters between two directions.

	Toward the paretic side (*n* = 17)	Toward the nonparetic side (*n* = 17)	*P* value^a^
Turn time (sec)	6.11 (0.89)	6.54 (1.04)	0.063
TL (cm)	15.99 (8.31)	15.32 (7.10)	0.616
Turn speed (cm/sec)	2.55 (1.40)	2.36 (1.11)	0.415
Turn peak (sec)	0.86 (0.56)	1.34 (0.99)	0.147
Turn force (kg·m/s^2^)	67.04 (13.80)	71.56 (16.22)	0.144
Turn force/weight	1.01 (0.16)	1.09 (0.22)	0.139
Turn peak/time	0.14 (0.09)	0.21 (0.17)	0.229

Back time (sec)	5.76 (0.77)	5.51 (0.42)	0.239
BL (cm)	15.76 (8.29)	14.96 (7.08)	0.520
Back speed (cm/sec)	2.84 (1.56)	2.74 (1.32)	0.709
Back peak (sec)	1.62 (0.77)	1.74 (0.58)	0.546
Back force (kg·m/s^2^)	66.99 (13.67)	68.22 (13.48)	0.387
Back force/weight	1.01 (0.16)	1.03 (0.14)	0.363
Back peak/time	0.27 (0.11)	0.32 (0.10)	0.209

Data are presented by mean and standard deviation.

^a^The analyses were performed based on the paired *t*-tests for the averaged turning ability parameters of the 10 measurements of each subjects.

Turn time: the time of turning; TL: distance between the centers of pressure before and after turning; turn speed: the speed of turning; turn peak: the time to peak counteraction; turn force: peak counteraction of turning; turn force/weight: the ratio of peak counteraction of turning to body weight; turn peak/time: the ratio of turn peak to turn time. Back time: time of turning back; BL: length of turning back of center of pressure; back speed: the speed of turning back; back peak time: the time of peak counteraction from turning back; back force, peak counteraction of turning back; back force/weight: the ratio of peak counteraction of turning back to body weight.

**Table 3:** Factors associated with turning ability parameters for rolling from supine to side lying position.

Dependent variables	Independent variables	Univariable models^a^		Multivariable models^a^	
		*β* (95% CI)	*P* value	*β* (95% CI)	*P* value
Turn time (sec)	Toward the nonparetic side versus toward the paretic side	0.427 (0.130, 0.725)	0.005^*∗*^		
	BSLE ≤ 3	0.220 (−0.609, 1.049)	0.603		
	BSUE ≤ 3	0.058 (−0.883, 0.999)	0.904		
	PASS ≤ 18	0.383 (−0.540, 1.307)	0.416		
	TCT ≤ 50	0.027 (−0.773, 0.827)	0.947		

TL (cm)	Toward the nonparetic side versus toward the paretic side	−0.670 (−1.364, 0.024)	0.058		
	BSLE ≤ 3	0.923 (−6.048, 7.893)	0.795		
	BSUE ≤ 3	−2.807 (−10.562, 4.948)	0.478		
	PASS ≤ 18	1.049 (−6.804, 8.902)	0.793		
	TCT ≤ 50	−1.797 (−8.430, 4.835)	0.595		

Turn speed (cm/sec)	Toward the nonparetic side versus toward the paretic side	−0.189 (−0.382, 0.005)	0.056		
	BSLE ≤ 3	0.111 (−1.020, 1.243)	0.847		
	BSUE ≤ 3	−0.403 (−1.665, 0.859)	0.531		
	PASS ≤ 18	0.313 (−0.954, 1.581)	0.628		
	TCT ≤ 50	−0.197 (−1.278, 0.884)	0.721		

Turn peak (sec)	Toward the nonparetic side versus toward the paretic side	0.480 (0.216, 0.744)	<0.001^*∗*^	0.480 (0.214, 0.745)	<0.001^*∗*^
	BSLE ≤ 3	0.402 (−0.018, 0.823)	0.061	0.296 (−0.150, 0.741)	0.193
	BSUE ≤ 3	0.429 (−0.050, 0.908)	0.079	0.292 (−0.210, 0.794)	0.253
	PASS ≤ 18	−0.319 (−0.817, 0.179)	0.209		
	TCT ≤ 50	−0.173 (−0.608, 0.261)	0.434		

Turn force (kg·m/s^2^)	Toward the nonparetic side versus toward the paretic side	4.515 (−1.087, 10.117)	0.114		
	BSLE ≤ 3	−2.298 (−15.557, 10.961)	0.734		
	BSUE ≤ 3	−1.233 (−16.210, 13.744)	0.872		
	PASS ≤ 18	−1.408 (−16.381, 13.565)	0.854		
	TCT ≤ 50	−5.965 (−18.383, 6.453)	0.346		

Turn force/weight	Toward the nonparetic side versus toward the paretic side	0.076 (−0.017, 0.170)	0.108		
	BSLE ≤ 3	0.002 (−0.159, 0.163)	0.981		
	BSUE ≤ 3	0.076 (−0.102, 0.254)	0.403		
	PASS ≤ 18	−0.010 (−0.191, 0.172)	0.916		
	TCT ≤ 50	−0.022 (−0.176, 0.132)	0.778		

Turn peak/time	Toward the nonparetic side versus toward the paretic side	0.069 (0.027, 0.110)	0.001^*∗*^	0.069 (0.027, 0.111)	0.001^*∗*^
	BSLE ≤ 3	0.063 (0.000, 0.126)	0.052	0.018 (−0.043, 0.079)	0.559
	BSUE ≤ 3	0.063 (−0.010, 0.135)	0.092	0.085 (0.013, 0.157)	0.021^*∗*^
	PASS ≤ 18	−0.064 (−0.136, 0.009)	0.087	−0.089 (−0.156, −0.023)	0.009^*∗*^
	TCT ≤ 50	−0.036 (−0.100, 0.029)	0.282		

^a^The analyses were performed based on generalized linear models with generalized estimating equation.

Turn time: the time of turning; TL: distance between the centers of pressure before and after turning; turn speed: the speed of turning; turn peak: the time to peak counteraction; turn force: peak counteraction of turning; turn force/weight: the ratio of peak counteraction of turning to body weight; turn peak/time: the ratio of turn peak to turn time; ^*∗*^ *P* < 0.05.

**Table 4:** Factors associated with turning ability parameters for rolling back from side lying position to supine.

Dependent variables	Independent variables	Univariable models^a^		Multivariable models^a^	
		*β* (95% CI)	*P* value	*β* (95% CI)	*P* value
Back time (sec)	Toward the nonparetic side versus toward the paretic side	−0.252 (−0.496, −0.008)	0.043^*∗*^	−0.252 (−0.497, −0.007)	0.044^*∗*^
	BSLE ≤ 3	0.536 (0.188, 0.884)	0.003^*∗*^	0.536 (0.187, 0.885)	0.003^*∗*^
	BSUE ≤ 3	0.060 (−0.424, 0.545)	0.807		
	PASS ≤ 18	−0.213 (−0.688, 0.263)	0.381		
	TCT ≤ 50	−0.245 (−0.641, 0.151)	0.225		

BL (cm)	Toward the nonparetic side versus toward the paretic side	−0.797 (−1.482, −0.112)	0.023^*∗*^		
	BSLE ≤ 3	1.407 (−5.602, 8.416)	0.694		
	BSUE ≤ 3	−2.782 (−10.603, 5.040)	0.486		
	PASS ≤ 18	0.429 (−7.501, 8.358)	0.916		
	TCT ≤ 50	−1.683 (−8.376, 5.011)	0.622		

Back speed (cm/sec)	Toward the nonparetic side versus toward the paretic side	−0.096 (−0.240, 0.048)	0.193		
	BSLE ≤ 3	0.082 (−1.216, 1.380)	0.901		
	BSUE ≤ 3	−0.567 (−2.005, 0.872)	0.440		
	PASS ≤ 18	0.124 (−1.338, 1.586)	0.868		
	TCT ≤ 50	−0.271 (−1.508, 0.965)	0.667		

Back peak (sec)	Toward the nonparetic side versus toward the paretic side	0.129 (−0.161, 0.419)	0.384		
	BSLE ≤ 3	0.530 (0.085, 0.975)	0.020^*∗*^	0.492 (0.159, 0.825)	0.004^*∗*^
	BSUE ≤ 3	−0.061 (−0.636, 0.514)	0.836		
	PASS ≤ 18	−0.568 (−1.077, −0.059)	0.029^*∗*^	−0.079 (−0.542, 0.384)	0.738
	TCT ≤ 50	−0.630 (−1.017, −0.242)	0.001^*∗*^	−0.569 (−0.958, −0.181)	0.004^*∗*^

Back force (kg·m/s^2^)	Toward the nonparetic side versus toward the paretic side	1.234 (0.434, 2.033)	0.002^*∗*^		
	BSLE ≤ 3	−6.179 (−18.644, 6.286)	0.331		
	BSUE ≤ 3	−2.858 (−17.221, 11.505)	0.697		
	PASS ≤ 18	−2.999 (−17.356, 11.358)	0.682		
	TCT ≤ 50	−8.632 (−20.186, 2.922)	0.143		

Back force/weight	Toward the nonparetic side versus toward the paretic side	0.022 (0.009, 0.035)	0.001^*∗*^		
	BSLE ≤ 3	−0.063 (−0.199, 0.072)	0.359		
	BSUE ≤ 3	0.052 (−0.102, 0.206)	0.509		
	PASS ≤ 18	−0.032 (−0.188, 0.123)	0.683		
	TCT ≤ 50	−0.063 (−0.193, 0.066)	0.337		

Back peak/time	Toward the nonparetic side versus toward the paretic side	0.044 (−0.003, 0.091)	0.064	0.044 (−0.003, 0.092)	0.066
	BSLE ≤ 3	0.067 (−0.004, 0.137)	0.063	0.058 (0.005, 0.111)	0.032^*∗*^
	BSUE ≤ 3	−0.016 (−0.103, 0.071)	0.719		
	PASS ≤ 18	−0.099 (−0.172, −0.026)	0.008^*∗*^	−0.036 (−0.109, 0.038)	0.344
	TCT ≤ 50	−0.096 (−0.154, −0.037)	0.001^*∗*^	−0.076 (−0.137, −0.014)	0.017^*∗*^

^a^The analyses were performed based on generalized linear models with generalized estimating equation.

Back time: time of turning back; BL: length of turning back of center of pressure; back speed: the speed of turning back;

Back peak: the time of peak counteraction from turning back; back force, peak counteraction of turning back; back force/weight: the ratio of peak counteraction of turning back to body weight. Back peak/time: the ratio of back peak to back time; ^*∗*^ *P* < 0.05.

## References

[1] Bobath B. (1979). The application of physiological principles to stroke rehabilitation. *The Practitioner*.

[2] Bohannon R. W. (2007). Muscle strength and muscle training after stroke. *Journal of Rehabilitation Medicine*.

[3] Morris J. H., Van Wijck F., Joice S., Donaghy M. (2013). Predicting health related quality of life 6 months after stroke: the role of anxiety and upper limb dysfunction. *Disability and Rehabilitation*.

[4] Tanaka S., Hachisuka K., Ogata H. (1997). Trunk rotatory muscle performance in post-stroke hemiplegic patients. *American Journal of Physical Medicine & Rehabilitation*.

[5] Tanaka S., Hachisuka K., Ogata H. (1998). Muscle strength of trunk flexion-extension in post-stroke hemiplegic patients. *American Journal of Physical Medicine & Rehabilitation*.

[6] Cameron D. M., Bohannon R. W., Garrett G. E., Owen S. V., Cameron D. A. (2003). Physical impairments related to kinetic energy during sit-to-stand and curb-climbing following stroke. *Clinical Biomechanics*.

[7] Duarte E., Marco E., Muniesa J. M. (2002). Trunk control test as a functional predictor in stroke patients. *Journal of Rehabilitation Medicine*.

[8] Nitz J., Gage A. (1995). Post stroke recovery of balanced sitting and ambulation ability. *The Australian Journal of Physiotherapy*.

[9] Di Monaco M., Trucco M., Di Monaco R., Tappero R., Cavanna A. (2010). The relationship between initial trunk control or postural balance and inpatient rehabilitation outcome after stroke: a prospective comparative study. *Clinical Rehabilitation*.

[10] Chiang S.-L., Lin C.-H., Chang S.-T. (2014). Measurement of bed turning and comparison with age, gender, and body mass index in a healthy population: application of a novel mobility detection system. *BioMed Research International*.

[11] Kafri M., Dickstein R. (2005). Activation of selected frontal trunk and extremities muscles during rolling from supine to side lying in healthy subjects and in post-stroke hemiparetic patients. *NeuroRehabilitation*.

[12] Richter R. R., VanSant A. F., Newton R. A. (1989). Description of adult rolling movements and hypothesis of developmental sequences. *Physical Therapy*.

[13] Fedrizzi E., Pagliano E., Marzaroli M. (2000). Developmental sequence of postural control in prone position in children with spastic diplegia. *Brain & Development*.

[14] Fedrizzi E., Facchin P., Marzaroli M. (2000). Predictors of independent walking in children with spastic diplegia. *Journal of Child Neurology*.

[15] Hollands M. A., Sorensen K. L., Patla A. E. (2001). Effects of head immobilization on the coordination and control of head and body reorientation and translation during steering. *Experimental Brain Research*.

[16] Gjelsvik B., Breivik K., Verheyden G., Smedal T., Hofstad H., Strand L. I. (2012). The Trunk Impairment Scale—modified to ordinal scales in the Norwegian version. *Disability and Rehabilitation*.

[17] Verheyden G., Ashburn A., Burnett M., Littlewood J., Kunkel D. (2012). Investigating head and trunk rotation in sitting: a pilot study comparing people after stroke and healthy controls. *Physiotherapy Research International*.

[18] Hacmon R. R., Krasovsky T., Lamontagne A., Levin M. F. (2012). Deficits in intersegmental trunk coordination during walking are related to clinical balance and gait function in chronic stroke. *Journal of Neurologic Physical Therapy*.

[19] Lamontagne A., Paquet N., Fung J. (2003). Postural adjustments to voluntary head motions during standing are modified following stroke. *Clinical Biomechanics*.

[20] Naik S. K., Patten C., Lodha N., Coombes S. A., Cauraugh J. H. (2011). Force control deficits in chronic stroke: grip formation and release phases. *Experimental Brain Research*.

[21] Verheyden G., Vereeck L., Truijen S. (2006). Trunk performance after stroke and the relationship with balance, gait and functional ability. *Clinical Rehabilitation*.

[22] Hollands M. A., Patla A. E., Vickers J. N. (2002). ‘Look where you're going!’: gaze behaviour associated with maintaining and changing the direction of locomotion. *Experimental Brain Research*.

[23] Collin C., Wade D. (1990). Assessing motor impairment after stroke: a pilot reliability study. *Journal of Neurology Neurosurgery and Psychiatry*.

[24] Duarte E., Marco E., Muniesa J. M., Belmonte R., Aguilar J. J., Escalada F. (2010). Early detection of non-ambulatory survivors six months after stroke. *NeuroRehabilitation*.

[25] Benaim C., Pérennou D. A., Villy J., Rousseaux M., Pelissier J. Y. (1999). Validation of a standardized assessment of postural control in stroke patients: the Postural Assessment Scale for Stroke patients (PASS). *Stroke*.

[26] Hsieh C.-L., Sheu C.-F., Hsueh I.-P., Wang C.-H. (2002). Trunk control as an early predictor of comprehensive activities of daily living function in stroke patients. *Stroke*.

[27] Franchignoni F. (2003). Psychometric properties and practical attributes of the trunk control test in stroke patients. *Journal of Rehabilitation Medicine*.

[28] Wang C.-H., Hsueh I.-P., Sheu C.-F., Hsieh C.-L. (2005). Discriminative, predictive, and evaluative properties of a trunk control measure in patients with stroke. *Physical Therapy*.

[29] Verheyden G., Nieuwboer A., Mertin J., Preger R., Kiekens C., De Weerdt W. (2004). The Trunk Impairment Scale: a new tool to measure motor impairment of the trunk after stroke. *Clinical Rehabilitation*.

